# Prevalence of Cam and Pincer Deformities in the X-Rays of Asymptomatic Individuals

**DOI:** 10.1155/2017/8562329

**Published:** 2017-12-18

**Authors:** Steffen Thier, Daniel Gerisch, Christel Weiss, Stefan Fickert, Alexander Brunner

**Affiliations:** ^1^Orthopedic and Trauma Surgery Center, University Medical Centre Mannheim, University of Heidelberg, Theodor-Kutzer-Ufer 1-3, 68167 Mannheim, Germany; ^2^Institute of Biostatistics, Medical Faculty Mannheim, University of Heidelberg, Ludolf-Krehl-Straße 13-17, 68167 Mannheim, Germany; ^3^Sporthopaedicum Straubing, Bahnhofplatz 27, 94315 Straubing, Germany; ^4^Department of Orthopaedic Surgery, Medical University Innsbruck, Anichstrasse 35, 6020 Innsbruck, Austria

## Abstract

**Objective:**

The presence of radiological signs of femoroacetabular impingement (FAI) is not necessarily associated with symptoms. Hence, the prevalence of cam and pincer deformities in the overall population may be underestimated. The purpose of this study was to screen an unselected cohort of people without hip symptoms for native radiological signs of cam and pincer deformities to determine their actual prevalence.

**Materials and Methods:**

110 asymptomatic patients had AP pelvis X-rays and cross-table hip X-rays performed. We evaluated the images for the presence of cross-over signs and measured lateral center edge (LCE) angles, alpha angles (*α*-angles), and femoral offset ratios.

**Results:**

Positive cross-over signs were seen in 34%; LCE angles > 40° in 13%; and femoral offset ratios < 0.18 in 43%. In 41% of the patients, *α*-angles were >50°. Male patients showed significantly higher *α*-angles, lower offset ratios, and a higher prevalence of cross-over signs. In contrast, female patients had significantly higher LCE angles.

**Conclusion:**

According to our data, radiological signs of cam and pincer deformities are common in asymptomatic people. In clinical practice, patients presenting with hip pain and radiological signs of FAI should undergo further diagnostic evaluation. However, in asymptomatic patients, no further evaluation is recommended.

## 1. Introduction

During the last decade, femoroacetabular impingement (FAI) has been commonly recognized as a cause of chronic hip pain and a possible predisposing condition for the development of early osteoarthritis [1–5]. During internal rotation and flexion of the hip, repetitive abutment between the femoral neck junction and the acetabular rim may lead to a cascade of degenerative changes including labrum disruption, chondromalacia, and peripheral acetabular cartilage delamination [1, 6, 7]. In general, FAI may be caused by two major types of anatomic abnormalities. Cam-type FAI is characterized by an aspherical configuration of the femoral head-neck junction, and pincer-type FAI is associated with acetabular overcoverage or retroversion. Interestingly, several studies have shown that most patients actually have a combined form (mixed FAI) of the two types [4, 6–11].

However, the presence of these anatomic abnormalities is not necessarily associated with clinical symptoms. In daily life, a considerable percentage of people with cam or pincer deformities may not feel any pain, and their anatomic pathology may remain undetected. Recently, a number of studies have been performed to evaluate the prevalence of cam or pincer deformities in asymptomatic people. Hack et al. reported a prevalence of 14% after screening 200 volunteers from their hospital staff using MRI [12]. Likewise, Reichenbach et al. evaluated a cohort of asymptomatic recruits with MRI and found a prevalence of 24% [13]. In regular participants of high impact sports (such as football, ice hockey, or skiing), even higher percentages—between 55% and 87%—have been reported [14–16].

Most of the studies that have screened populations for cam or pincer deformities used MRI or CT scans. However, in daily clinical practice, patients who present with hip pain will first have native X-rays obtained [4, 6, 17, 18]. To improve the interpretation accuracy of these X-rays, it may be helpful to know the prevalence of deformities associated with FAI on plain films in the average asymptomatic population. Until recently, studies evaluating X-rays of asymptomatic people for cam and pincer deformities were limited. Therefore, the purpose of this study was to screen an unselected cohort of people without hip symptoms for native radiological signs associated with cam and/or pincer FAI. We hypothesized that a significant percentage of asymptomatic people would have radiological signs of FAI on native X-rays.

## 2. Material and Methods

This retrospective study was approved by the Ethics Committee of the Medical University Heidelberg (Process number 2011-370N-MA) and is in accordance with the declaration of Helsinki. Between 2010 and 2011, AP pelvis and cross-table radiographs were routinely performed for patients who presented to our emergency department with acute trauma to the hip or femur. The X-rays and medical files of these patients were retrospectively evaluated.

Inclusion criteria for the study included the presence of AP pelvis and cross-table radiographs of adequate quality, information on the patient's medical history, and patient age greater than 18 years.

Patients who reported having hip symptoms (e.g., pain or limited range of motion) before the presenting trauma and those who sustained femoral or acetabular fractures during the presenting trauma were excluded. Further exclusion criteria included prior hip surgery, a history of hip fracture, congenital hip pathologies (dysplasia, Legg-Calve-Perthes disease, and slipped capital femoral epiphysis), grade IV osteoarthritis on X-ray according to the Kellgren–Lawrence scale [19], and neurological pathologies that could affect physiological loading of the hip.

Finally, 110 patients (60 women, 50 men) with 114 hips (60 left, 54 right) and a mean age of 56 years (range: 18–100 years, SD: 22.4 years) were included in this study.

### 2.1. X-Rays

Standard AP pelvic radiographs were performed with the patients in supine position and the hip extended and internally rotated 15°. The film-focus distance was 1.2 meters, with the central beam directed toward the intersection of the line connecting both anterior-superior iliac spines and a vertical line through the symphysis [2, 22].

X-ray quality was considered adequate when both obturator foramen and iliac crests were symmetrically projected, and the coccyx was projected 1-2 cm caudal to the symphysis [2].

Cross-table lateral radiographs were performed with the patient in the supine position and the hip extended and internally rotated 15°. The contralateral hips and knees were flexed beyond 80°. The central X-ray beam was positioned parallel to the ground with an inclination of 65°, according to the axis of the femur, and directed toward the inguinal fold [7, 23].

### 2.2. Radiological Evaluation

**Figure 1:**
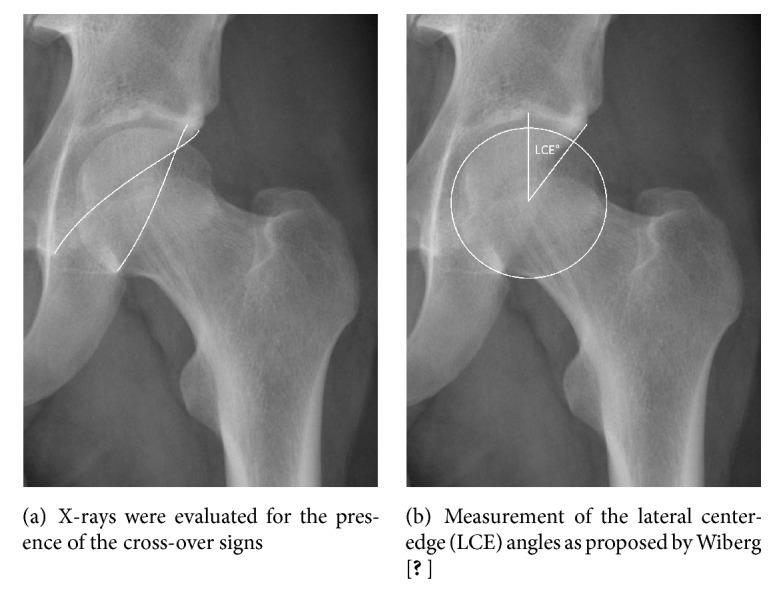


**Figure 2:**
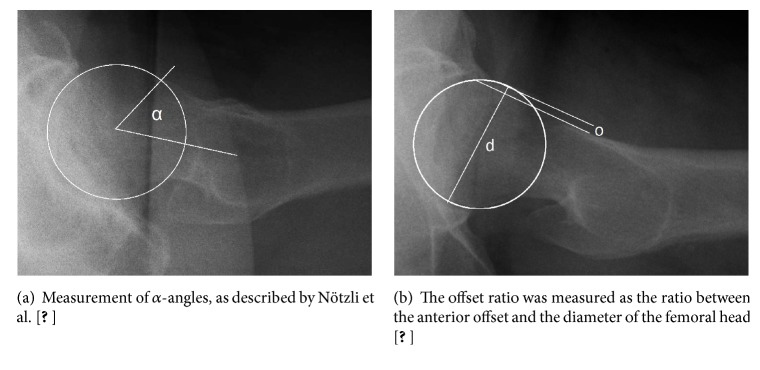


Radiographs were assessed on a SYNGO-Viewer workstation (Siemens Healthcare, Erlangen, Germany). A third person not involved further in the evaluations removed patient data from the X-rays. To assess the prevalence of pincer deformities, two orthopaedic surgery consultants independently evaluated the AP pelvis X-rays in random order for the presence of the cross-over signs (Figure 1(a)) [8, 11, 24] and measured the lateral center-edge (LCE) angles as proposed by Wiberg (Figure 1(b)) [20]. A LCE angle of 40° or higher was considered to represent a pincer deformity. To assess the prevalence of cam deformities, cross-table lateral radiographs were evaluated for femoral asphericity by the measurement of *α*-angles (Figure 2(a)), as described by Nötzli et al. [21], and by the ratio between the anterior offset and the diameter of the femoral head (i.e., the offset ratio; Figure 2(b)). An offset ratio < 0.18 was considered pathological [22]. Finally, all radiographs were assessed for grade of osteoarthritis according to the Kellgren–Lawrence grading scale [19].

Mean values of the measured parameters (LCE angles, *α*-angles, and offset ratios) were used for further analyses. In cases with inconsistencies regarding the presence of a cross-over signs or the grade of osteoarthritis, X-rays were reevaluated by both orthopaedic surgeons together and a consensus decision was made.

### 2.3. Statistical Analysis

A Shapiro–Wilk analysis was performed to test linear data for normal distribution. We calculated mean values for linear data. The number of patients with positive cross-over signs, LCE angles ≥ 40°, and offset ratios < 0.18 were calculated. Student's *t*-test was used to compare mean values for linear data, and the chi-square test was used to compare frequencies for nominal data. Since the *α*-angle cut-off considered pathological varies in the literature, we calculated the number of patients with *α*-angles >50°, >58°, >62°, >70°, and >83°. A Pearson correlation analysis was performed to assess for correlations among *α*-angles, LCE angles, offset ratios, patient age, and the grade of osteoarthritis. A correlation coefficient (*r*) < 0.3 was considered to indicate a weak correlation; 0.3–0.7, a moderate correlation; and >0.7, a high correlation. A *p* value < 0.05 was considered statistically significant.

## 3. Results

Shapiro–Wilk analyses showed significant normal distributions for patient age (*p* < 0.01), *α*-angles (*p* < 0.01), and offset ratios (*p* = 0.01). There were 33 hips (13 men, 17 women) without signs of OA (Kellgren–Lawrence grade 0). However, 20 hips (11 men, 9 women) showed OA grade 1; 37 hips (14 men, 22 women), OA grade 2; and 24 hips (12 men, 12 women), OA grade 3. There were 39 patients (39 hips; 25 men, 14 women; 34% of all hips) with a positive cross-over sign. The prevalence of cross-over signs was significantly higher in male patients than in female patients (*p* < 0.01).

The mean LCE angle was 30.7° (range: 13°–52°), and the mean offset ratio was 0.18 (range: −0.07–0.33; Table 1). There were 15 patients (13%; 5 men, 10 women) with LCE angles ≥ 40° and 49 patients (43%; 30 men, 19 women) with an offset ratio < 0.18. Female patients showed significantly higher LCE angles compared with males (*p* = 0.01). In contrast, male patients showed significantly lower offset ratios compared with females (*p* < 0.01; Table 1).

**Figure 3:**
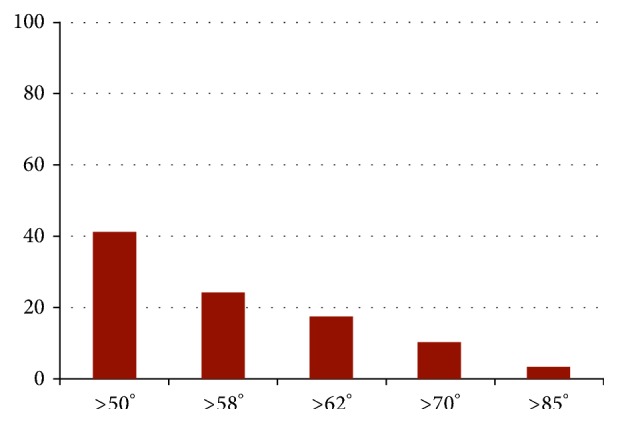
The percentage of patients with *α*-angles exceeding different cut-off values.

The mean *α*-angle was 50.9° (range: 33°–89°). There were 47 patients (30 men, 17 women) with *α*-angles > 50° (mean: 69.5°; range: 51°–89°); 28 patients (21 men, 7 women) with *α*-angles > 58° (mean: 63.3°; range: 59°–89°); and 4 patients (2 men, 2 women) with *α*-angles > 83° (mean: 63.3°; range: 84°–89°).  Figure 3 shows the percentage of patients with *α*-angles exceeding different cut-off values. Male patients had significantly higher *α*-angles compared with females (*p* < 0.01).

Correlation analysis showed a moderate correlation between LCE angles and patient age (*r* = 0.365, *p* < 0.01). No correlation was found between age and offset ratios (*r* = 0.169, *p* = 0.07) or between age and *α*-angles (*r* = −0.131, *p* = 0.17).

Similarly, no significant correlation was found between the stage of osteoarthritis and LCE angles (*r* = 0.176, *p* = 0.06), offset ratios (*r* = −0.85, *p* = 0.37), or *α*-angles (*r* = 0.105, *p* = 0.26).

In summary, 82 patients (71.9%) showed at least one radiological sign of cam or pincer deformity, 52 patients (45.6%) showed two, and 18 patients (15.8%) showed three; none of the patients showed more than three signs (Tables 2, 3, and 4).

## 4. Discussion

These data confirm our hypothesis that a number of asymptomatic people would have signs of cam and pincer deformities on native hip X-rays. This is in accordance with data from studies that have reported FAI signs on hip X-rays of 42.6% to 53% of asymptomatic individuals [23, 25, 26].

The mechanical basis of FAI is the result of a complex, dynamic interaction between the femoral head-neck junction and the acetabular labrum or rim [27, 28], which is difficult to assess using two-dimensional imaging techniques. In recent years, assessment of cam deformities has often been performed by measuring *α*-angles at the anterior aspect of the femoral head-neck junction [5, 21, 29–31]. However, several studies have shown that the area of maximum extension of the cam deformity may vary between patients and that the greatest loss of head-neck asphericity is present at either the anterior aspect of the head-neck junction or the anterior-superior surface [32–34]. As a result, a straight axial X-ray of the femoral head-neck junction may underproject the maximum extension of the cam deformity in some cases. Likewise, standard anteroposterior X-rays do generally not project the deformity leading to cam FAI [35–37]. Consequently, several native X-ray views have been proposed to measure the *α*-angle, such as the frog-leg lateral view [17], the 45° and 90° Dunn views [38], and the cross-table lateral view [18]. Meyer et al. [35] evaluated a number of native X-ray views and found the highest sensitivity in cam deformity detection for the 45° Dunn view and the cross-table view in 15° internal rotation. Since the cross-table view showed the highest inter- and intraobserver reliability [35], we decided to use it for our study. A number of studies [23, 25, 26] have evaluated cam deformities in asymptomatic people on the basis of 45° Dunn views, but studies using internally rotated cross-table views remain sparse.

However, according to the complex, three-dimensional interaction between femoral head-neck junction and acetabular rim, the area with the maximum deformity is not necessarily the area that causes the impingement. Therefore, it is difficult to define a clear threshold when to consider a deformity pathological. Sole measurement of the deformities maximum extension may result in overestimation of the pathology.

In general, the data from this study are consistent with findings from several CT- and MRI-based studies [12, 13, 29, 31] that have reported the prevalence of cam deformities in asymptomatic patients to be between 10% and 31%. In contrast to cam FAI, which seems clearly associated with a loss of the femoral head-neck offset [21], the pincer type is more difficult to assess [1, 29, 39]. A prior study has shown a correlation between LCE angles on plain AP X-rays and acetabular overcoverage [40]. Likewise, the cross-over sign has shown a high sensitivity and specificity in detecting acetabular retroversion [29]. In contrast, recent studies have questioned the value of these two measures in the assessment of pincer deformities [24, 41]. Isolated coxa profunda seems to rarely be associated with pincer FAI [39].

In our study, 13% of patients had an LCE angle > 40°, which is comparable to the 14% reported by Diesel et al. [39]. In contrast, 34% of the asymptomatic patients in our study showed positive cross-over signs, which is higher than the 12.6%–18.5% reported from previous research [25, 26, 39]. Signs of pincer impingement were significantly more frequent in females, and cam deformities were significantly more frequent in males. No correlation between age and *α*-angle was found. These observations are in line with results from several other studies [25, 30, 34, 39].

What remains unclear is whether asymptomatic people with cam or pincer deformities do not actually impinge, or if they remain asymptomatic because the impingement occurs without causing pain. Furthermore, it is unknown if patients with painless FAI are more likely to develop early OA compared with people without anatomical deformities [42, 43].

A recent meta-analysis evaluated the role of prophylactic surgery for asymptomatic FAI [44]. Considering the lack of available evidence and known complication rates as high as 6.4% after arthroscopic surgery [45], prophylactic surgery could not be recommended for asymptomatic patients with FAI [44].

The major limitations of the current study were the relatively small sample size and its retrospective design.

## 5. Conclusion

According to our data, radiological signs of cam and pincer FAI are frequently found in asymptomatic individuals. In clinical practice, patients presenting with hip pain and native radiological signs of FAI should undergo further diagnostic evaluation. In asymptomatic patients with radiological signs of FAI, however, no further diagnostic studies are recommended.

## Figures and Tables

**Table 1:** Mean LCE angles, *α*-angles, and offset ratios for male patients, female patients, and all patients. The right column shows *p* values for comparisons between men and women.

	Male patients (*n* = 53)	Female patients (*n* = 61)	Mean (*n* = 114)	Stat. difference between male/female patients
LCE angles (range)	28.9° (13°–52°)	32.3° (21°–46°)	30.7° (13°–52°)	*p* = 0.01
Offset ratio	0.15 (−0.07–0.31)	0.20 (−0.06–0.33)	0.18 (−0.06–0.33)	*p* < 0.01
*α*-Angles	54.7° (35°–89°)	47.6° (33°–88°)	50.9° (33°–89°)	*p* < 0.01

**Table 2:** Overall prevalence of FAI signs.

Signs of FAI	Prevalence in % (number of pat.)
1	71.9% (82/114)
2	45.6% (52/114)
3	15.8% (18/114)
4	0

**Table 3:** Prevalence of CAM-type FAI signs.

Signs of FAI	Prevalence in % (number of pts.)
1	43.0% (49/114)
2	41.2% (47/114)

**Table 4:** Prevalence of pincer-type FAI signs.

Signs of FAI	Prevalence in % (number of pts.)
1	42.1% (48/114)
2	3.5% (4/114)
